# Application of Injectable Hydrogels as Delivery Systems in Spinal Cord Injury

**DOI:** 10.3390/gels9110907

**Published:** 2023-11-16

**Authors:** Rong Ji, Zhizhong Hao, Hao Wang, Xingfan Li, Linyan Duan, Fangxia Guan, Shanshan Ma

**Affiliations:** School of Life Sciences, Zhengzhou University, Zhengzhou 450001, China; jirong000@163.com (R.J.); lilachzz@icloud.com (Z.H.); 18236797424@163.com (H.W.); lixingfan0929@163.com (X.L.); duanlinyan4881@163.com (L.D.)

**Keywords:** injectable hydrogel, spinal cord injury, delivery systems, application, mechanism

## Abstract

Spinal cord injury (SCI) is a severe neurological injury caused by traffic accidents, trauma, or falls, which leads to significant loss of sensory, motor, and autonomous functions and seriously affects the patient’s life quality. Although considerable progress has been made in mitigating secondary injury and promoting the regeneration/repair of SCI, the therapeutic effects need to be improved due to drug availability. Given their good biocompatibility, biodegradability, and low immunogenicity, injectable hydrogels can be used as delivery systems to achieve controlled release of drugs and other substances (cells and proteins, etc.), offering new hope for SCI repair. In this article, we summarized the types of injectable hydrogels, analyzed their application as delivery systems in SCI, and further discussed the mechanisms of hydrogels in the treatment of SCI, such as anti-inflammatory, antioxidant, anti-apoptosis, and pro-neurogenesis. Moreover, we highlighted the potential benefits of hydrogels in the treatment of SCI in combination with therapies, including the recent advances and achievements of these promising tools. Our review may offer new strategies for the development of SCI treatments based on injectable hydrogels as delivery systems.

## 1. Introduction

Spinal cord injury (SCI), one of the most serious public health problems, often results in irreversible neurological damage and dysfunction, which has huge emotional, economic, and social impacts on patients and their families [1]. The leading causes of SCI include collisions, sports-related accidents, falls, and violence-related injuries [2,3]. SCI can be divided into primary injuries and secondary injuries. Primary injuries occur at the initial stage of injuries and disrupt the structural integrity of the tissue within seconds, and they are characterized by tears in bone fragments and spinal ligaments. Secondary injuries are divided into three stages: acute, subacute, and chronic stage [4]. Acute secondary injuries are direct results of the primary mechanical trauma and are accompanied by features such as vascular damage, free radical production, lipid peroxidation, inflammation, and ischemia [5]. In the subacute phase, the events include cell apoptosis, axonal demyelinating, and glial scarring. Subacute secondary injury can lead to a chronic phase of SCI, characterized by cystic cavity formation and glial scarring maturation [4,6].

**Figure 1:**
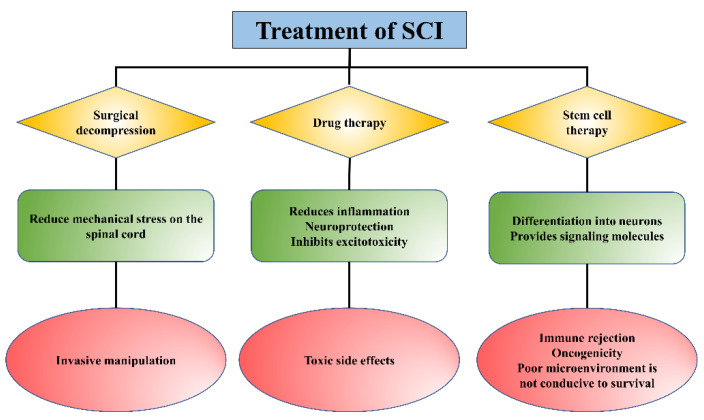
The role and limitations of existing treatments for SCI.

At present, the treatment methods for SCI mainly include surgical decompression, pharmacological intervention, and non-drug treatment (such as cell-based transplantation), etc. (Figure 1) [7,8,9,10,11]. Surgical decompression is to mitigate further secondary injury by reducing the mechanical stress on the spinal cord exerted by bleeding and edema [12]. However, surgical decompression is an invasive method with no uniform standard for surgical timing, and the timing of surgery remains controversial for patients. Methylprednisolone, riluzole, phenylpropylpyridine, erythropoietin (EPO), and minocycline are common clinical drugs for SCI therapy to reduce the inflammatory response at the injury area and inhibit excitotoxicity, but there are some limitations, such as side effects and other complications [13,14]. Cell therapies, represented by stem cells, may be promising strategies for the treatment of SCI [9]. Due to their multidirectional differentiation potential and immunomodulatory and active factor secretion functions, stem cells have become the seed cells and have been widely used in SCI therapy with many breakthroughs [15]. The cell types commonly used for SCI treatment include embryonic stem cells (ESCs), induced pluripotent stem cells (iPSCs), mesenchymal stem cells (MSCs), neural stem cells (NSCs), and others [16]. However, the harsh microenvironment in the lesion is not conducive to the survival, proliferation, and differentiation of the implanted stem cells [17]. Thus, although considerable progress has been made in the treatment and care of SCI, clinical outcomes are less than satisfactory, and there is an urgent need to develop new strategies for the treatment of SCI.

Flourishing advances of biomaterials and tissue engineering technologies have provided novel therapeutic strategies for SCI treatment [1,18]. Hydrogels are a kind of highly hydrated polymeric materials with three-dimensional network structures that can maintain their structural integrity through physical and chemical interactions [19]. As a multifunctional platform, the physical and chemical properties of hydrogels (e.g., hardness, pore size, viscoelasticity, degradability, and stimulus-responsive properties) can be tuned through rational structural and functional design [20]. Thus, hydrogels are widely used in the treatment of refractory diseases, such as SCI, due to their injectability, good biocompatibility, biodegradability, and ability to match irregular injuries [21,22]. In addition, hydrogels can be used as delivery systems for cells, drugs, or other substances to achieve the long-term controlled release of drugs or cellular molecules [23,24,25], making them highly valuable for the treatment of SCI [26].

In this paper, we aimed to summarize the common types of hydrogels used in SCI therapy, reviewed the use of hydrogels as delivery systems in SCI, and highlighted the molecular mechanisms of hydrogel therapy for SCI. In addition, we focused on the effects of hydrogels in combination with other techniques for the repair of SCI. Our study will further contribute to the development of hydrogel as a delivery system for the treatment of SCI and provide strategies for SCI repair.

## 2. Types of Hydrogels

According to the source of materials, hydrogels can be divided into natural hydrogels, synthetic hydrogels, and natural–synthetic composite hydrogels (Table 1), and the different types of hydrogels will be introduced below [27].

### 2.1. Natural Hydrogel

Natural hydrogels are formed by the polymerization of natural biomaterials or their derived derivatives, such as hyaluronic acid (HA), collagen, chitosan, agarose, alginate, etc. [52]. Natural polymers have inherent advantages, including abundant natural availability, specific molecules for cell adhesion, biodegradable, and biocompatibility [53], which can also reduce chronic inflammation or stimulation of the immune response due to their similarity to extracellular matrix (ECM) [54]. The various advantages of natural hydrogels make them widely used in the treatment of SCI. Kushchayev et al. found that HA hydrogel could protect the spinal cord from inflammation and reduce secondary injury in a SCI model of Sprague–Dawley (SD) rats [28], and injectable hyaluronate hydrogels with rapid self-healing ability served as a bridge and promoted angiogenesis, remyelination, and neural regeneration in SCI mice [29]. In addition, implantation of HA hydrogel in a rat model of dorsal hemisection injury of the spinal cord limited astrocyte activation and scar formation [30]. Collagen is highly abundant in the ECM and is an auspicious scaffold for promoting the repair, recovery, and regeneration of SCI [55]. Collagen hydrogel combined with small molecules, stem cells, or exosomes could promote neurogenesis, inhibit cell apoptosis and reduce glial scar production in SCI sites [31,32,33]. Additionally, some in vitro experiments were also carried out on collagen hydrogels before animal experiments, which confirmed that the prepared collagen scaffold has very good biocompatibility; does not affect the adhesion, growth, and differentiation of NSCs in vitro; and is not cytotoxic, so further animal experiments can be carried out [33].

Chitosan has excellent biological properties, such as nontoxicity, biodegradation, and antibacterial activity [56]. Recently, chitosan-based hydrogel has attracted significant attention for SCI repair in nerve tissue engineering applications, which could inhibit neuroinflammation, promote the recovery of motor function, and prevent scar tissue formation in SCI animals [34,35,36]. In addition, Han et al. indicated that agarose scaffold containing Matrigel could support and enhance the regeneration of damaged spinal axons, and successfully reestablish the descending motor projection between motor cortical neurons and outside the lesion site [37]. Moreover, alginate is a natural biocompatible hydrogel extracted from brown seaweed, and alginate hydrogel could promote spinal cord neural stem/progenitor cell differentiation and locomotor recovery after SCI [38,39,57]. Huang et al. also showed that alginate hydrogel significantly improved both electro-physiological conductivity and motor function and promoted axonal growth in the cross-section of the chronic spinal cord after scar removal [40]. Moreover, the implantation of agarose hydrogel scaffolds could promote nerve regeneration after SCI and encapsulated neurotrophic factors to reduce inflammation at the site of injury [41].

Collectively, natural hydrogels are efficient scaffolds for the functional recovery of SCI and even a much better scaffold for drug/cell delivery after SCI [58,59]. However, the potential of natural hydrogels in SCI therapy may be limited by their long-term stability and degradation. Although these natural hydrogels have no significantly toxicity in cellular and animal studies, their safety and potential risk to SCI patients have not been evaluated in clinical trials, suggesting the fundamental need for standardized purification and toxicity studies prior to clinical use.

### 2.2. Synthetic Hydrogel

Synthetic hydrogels are formed through the physical or chemical cross-linking of synthetic polymers. Synthetic polymers are easier to mass-produce and have highly adjustable properties that can be customized to the needs of specific applications [60]. Polyethylene glycol (PEG), polyacrylamide (PAM), polyhydroxyethyl methacrylate (PHEMA), and poly-ε-caprolactone (PCL) are the common synthetic polymers [27]. PEG is a versatile polymer with no apparent toxicity or irritation [61], and hydrophilic PEG hydrogels can be fabricated through multiple crosslinking to produce scaffolds with different degradation rates and drug release rates [62,63]. Accumulating evidence has shown that PEG hydrogel could inhibit neuroinflammation in the early stages of SCI, repair membrane damage, promote axonal regeneration, and improve motor function after severe SCI [42,64,65]. In addition, the superior chemical and mechanical properties of PEG hydrogels allow them to act as delivery vehicles for bioactive molecules, including growth factors (GFs) and cells, which modulate inflammatory responses and support neural tissue [66,67].

Additionally, PAM hydrogels are widely used in the field of peripheral nerve regeneration because of their good biocompatibility, [68] but pure PAM hydrogels usually have poor mechanical properties and high brittleness [69]. Jiang et al. prepared PAM gels using DNA as a crosslinker, and when rat spinal cord cells were cultured in the hydrogel for a period of time, spinal cord neurons were observed to extend primary dendrites and shorter axons on the gel [43]. PHEMA has high mechanical strength and good biostability, which can be used as a basic ingredient in the treatment of SCI as a hydrogel. Hejcl et al. found that positively charged HEMA hydrogels can bridge a posttraumatic spinal cord cavity and provide a scaffold for the ingrowth of regenerating axons [44]. Additionally, modified PHEMA hydrogel had bioadhesive properties that promoted tissue bridging as well as aligned axonal ingrowth at the site of injury [45,46]. PCL is biodegradable and biocompatible [70] which can be used as delivery carriers in tissue engineering. Terraf et al. indicated that PCL scaffolds seeded with human endometrial stem cells could restore the continuity of the damaged spinal cord and decreased cavity formation, contributing to the functional recovery of the spinal cord [47]. Babaloo et al. found that implantation of PCL/gelatin scaffolds loaded with endometrial stem cells could minimize secondary damage to the spinal cord, improve motor function, and lead to axonal remyelination, and endometrial stem cells (EnSCs) exhibited appropriate expansion and growth on PCL/gelatin scaffolds [48]. As a result, these synthetic polymers have promising applications and good results in tissue engineering, drug delivery, and SCI damage repair. However, the efficacy of synthetic hydrogels alone is not ideal [71]. Functionally modified synthetic hydrogels can to some extent reduce the toxicity of polymeric materials and expand the beneficial applications of synthetic hydrogels, which remains a tough challenge and a hot topic in tissue engineering.

### 2.3. Composite Hydrogel

Composite hydrogels combine the biocompatibility of natural hydrogels with the tunable mechanical and physical properties of synthetic hydrogels, thus holding promise for a wide range of applications in SCI [27]. Zhao et al. developed a double crosslinked biomimetic composite hydrogel comprised of acellularized spinal cord matrix, gelatin-acrylated-β-cyclodextrin-polyethene glycol diacrylate, and WAY-316606, which could recruit endogenous neural stem cells, improve neuronal differentiation, and induce neural tissue regeneration and functional recovery after SCI with no obvious cytotoxicity [49]. In addition, a novel multifunctional composite hydrogel consisting of fibrin hydrogels and functionalized self-assembling peptides promoted spinal cord regeneration by guiding regenerative tissue, accelerating axonal regeneration and remyelination, and promoting angiogenesis [50]. Additionally, Khaing et al. developed a highly versatile injectable composite hydrogel to sustained deliver brain-derived neurotrophic factor (BDNF) by using HA, methylcellulose (MC), and polylactic acid-glycolic acid (PLGA) microparticles. They found that this composite biomaterial system can be utilized for the sustained and localized delivery of therapeutics to benefit to the recovery of SCI [51]. Taken together, composite hydrogels offer multiple advantages that facilitate the functional recovery of SCI. The development of more effective composite functional hydrogels to improve the biocompatibility, safety, and efficacy of biomaterials is key to the treatment of SCI.

## 3. Application of Hydrogel as a Delivery System in SCI

**Figure 2:**
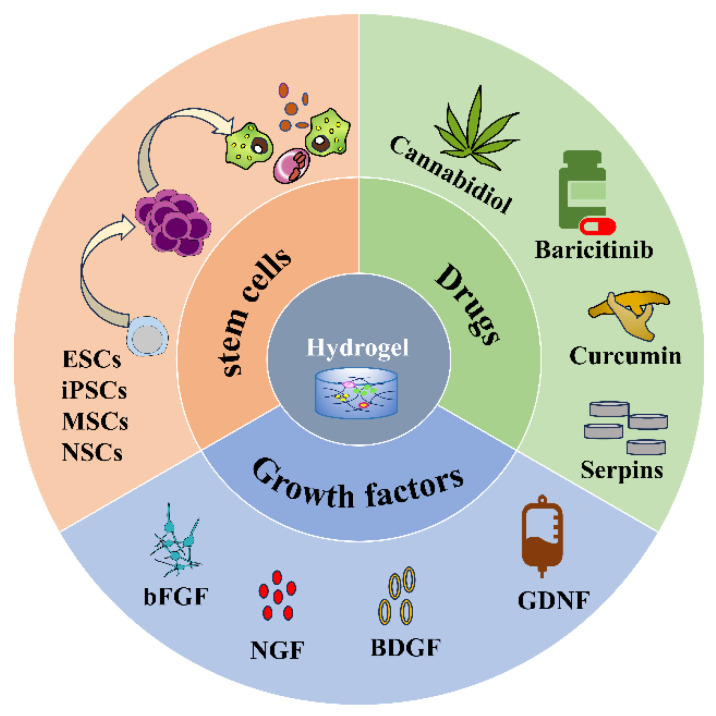
Hydrogel loaded with stem cells, drugs, and growth factors.

Injectable hydrogels as delivery systems can encapsulate different substances, such as cells, drugs, and biomolecules (Figure 2) and release them slowly and controllably at the site of injury to improve drug utilization and efficacy (Table 2). Thus, hydrogels have received accumulating attention and made many breakthroughs in the treatment of SCI as a drug delivery system.

### 3.1. Stem Cells

Stem cells are a kind of cells with the potential of self-renewal and multi-differentiation, which can repair damaged tissues, improve microenvironment, and promote tissue regeneration through substitution and paracrine and immune regulation [86]. Stem cell therapy has great application prospects in neurological diseases such as SCI. The cell types commonly used for SCI treatment include embryonic stem cells (ESCs), induced pluripotent stem cells (iPSCs), mesenchymal stem cells (MSCs), neural stem cells (NSCs), and others [16]. However, the efficacy of single stem cell transplantation cannot meet people’s expectations, mainly because the inflammatory and oxidative stress microenvironment at the injured site is not conducive to the survival and differentiation of stem cells [87,88]. Additionally, the implanted stem cells are easy lose with the blood, which makes it difficult for free stem cells to form functional networks, thus seriously affecting the efficacy of stem cells [9].

In recent years, researchers have used tissue engineering techniques to prepare a variety of hydrogel scaffolds loaded with stem cell grafts to achieve better results in neural repair due to the good biocompatibility and biodegradability of hydrogels [22]. In terms of SCI repair, hydrogel combined with stem cell therapy has three advantages: (1) The hydrogel can be used as a carrier to load stem cells to the lesion, reducing the loss of cells to surrounding tissues; (2) hydrogels provide 3D support, which is conducive to cell migration, proliferation, and differentiation; (3) functional hydrogels can regulate the microenvironment of the damaged site and protect the activity and biological function of stem cells [15,89]. For example, Huang et al. found that dual-network conductive hydrogels based on dextran promoted the differentiation of NSCs into neurons and inhibited the differentiation of astrocytes, suggesting that these hydrogels have great potential in SCI regeneration as biomimetic materials [72]. Collagen/HA scaffolds and collagen/nanofiber scaffolds can promote NSCs to differentiate into neuron-like cells and thus play neuroprotective roles [73]. Yao et al. found that dual-enzymatically cross-linked gelatin hydrogel could enhance the neural differentiation of human-umbilical-cord-derived MSCs and functional recovery in an experimental murine SCI model [74]. In 2022, Wertheim L et al. for the first time demonstrated that an ECM hydrogel loaded with human iPSCs promoted the efficient differentiation of iPSCs into spinal cord cells, induced the formation of spinal cord tissue, and then implanted into a mouse model of SCI-induced paralysis to make them regain their mobility [75]. Yuan et al. developed a cell-adaptive neurogenic (CaNeu) hydrogel as a delivery carrier of adipose-derived stem cells (ADSCs) [76]. CaNeu hydrogel dynamic networks loaded with ADSCs provided a cell-infiltrating matrix that promoted axon growth, reduced neuroinflammation, and ultimately improved motor function in SCI rats. Thus, these findings suggest that injectable hydrogels are a valuable delivery vehicle for stem cell therapy with no significant cytotoxicity, providing a promising strategy for the treatment of SCI [23].

Although great progress has been made in the treatment of SCI by combining hydrogels with stem cells, we still need to be cautious. The main causes include that: (1) Most of the in vivo studies were conducted in animal models, and human clinical trials should be an inevitable target for the application of hydrogels loaded with stem cells; (2) the selection of suitable hydrogel scaffolds and active factors is crucial for the preparation of neuro-functional scaffolds and the promotion of SCI function repair [15].

### 3.2. Drugs

Though many drugs have been tested in experimental models of SCI, the clinical translation of established therapeutic agents for the treatment of SCI remains challenging. First, most systemic drugs cannot reach the site of injury due to the impermeability of the blood–spinal cord barrier [90]. In addition, most drugs usually have short half-lives and require high doses and/or frequent administration to reach therapeutic concentrations at the site of injury, which can lead to harmful side effects and may lead to sustained inflammatory activation [91]. Injectable hydrogels with sustained drug delivery properties, degradability, and tunable physical properties can overcome and optimize these shortcomings either as a model of transport for the drug itself or as a carrier for drug-loaded particles/carriers [22,92]. Erythropoietin (EPO) is a growth factor that exhibits neuroprotective effects in the treatment of SCI [93]. Studies have shown that EPO-chitosan/alginate (EPO-CH/AL) hydrogels have controlled release characteristics for EPO, and EPO-CH/AL hydrogels significantly improve tissue repair and the histopathological appearance of the spinal cord at the site of injury [77]. Serine protease inhibitors (serpins) are “suicide” inhibitors with a highly conserved structure, which prevents excessive bleeding or clotting. Kwiecien et al. implanted chitosan–collagen hydrogel encapsulated with serpins at injury sites of SCI rats, which could improve neurological and motor functions and reduce tissue damage caused by inflammation in SCI rats [78]. Cannabidiol (CBD) is a non-psychotropic phytocannabinoid derived from cannabis that has anti-inflammatory, antioxidant, and neuroprotective effects [94]. Zhang et al. implanted CBD-loaded injectable chitosan hydrogel into SCI rats and observed that the hydrogel could sustain delivery of CBD, reduce apoptosis, and improve neurogenesis by enhancing mitochondrial biogenesis [79]. Baricitinib is a small inhibitor of Janus kinase (JAK) approved for the treatment of certain inflammatory diseases [95]. Zheng et al. have shown that an injectable PLGA-PEG-PLGA thermos-responsive hydrogels loaded with baricitinib could reduce neuronal apoptosis and promote functional recovery in SCI rats by inhibiting the JAK2-STAT3 pathway and decreasing neuroinflammation [80]. Moreover, curcumin has anti-inflammatory and antioxidant effects that may be neuroprotective in neurological injury. Luo et al. designed an injectable and self-healing hydrogel fabricated from chitosan with the controlled release of curcumin to repair SCI. They found that this composite hydrogel could reassemble ECM at the lesion site, participated in the remyelination process of the regenerated nerves, and favored functional recovery of SCI rats [81]. Taken together, hydrogels encapsulated with drugs (natural extracts, small molecule drugs, etc.) have promising application prospects in the treatment of SCI. However, suitable hydrogels need to be designed to achieve the controlled release of drugs at the site of injury and to reduce the toxicity of both drugs and hydrogels [26].

### 3.3. Growth Factors

Growth factors (GFs) could stimulate the growth of specific tissues, direct specific cellular responses in the microenvironment, and promote axonal regeneration [96]. Commonly used GFs include fibroblast growth factor (bFGF), nerve growth factor (NGF), BDNF, and glial neurotrophic factor (GDNF), etc., all of which are associated with neurodevelopment and neurogenesis [97]. The use of GFs for SCI has been shown to promote axonal regeneration and functional recovery [98]. However, direct administration of GFs is limited by their rapid degradation and dilution at the site of injury [82]. As a biocompatible biological scaffold, hydrogels have a high affinity for GFs and can stably control the release of GFs, avoiding the side effects of high GFs concentrations at the injection site and protecting them from enzymatic hydrolysis [99].

Thus, controlled delivery of multiple GFs to the lesion is becoming an attractive strategy for repairing SCI. For instance, Hu et al. developed a heparin-poloxamer (HP)-based hydrogel for the delivery of bFGF and NGF, which significantly improved neuronal survival, inhibited reactive astrogliosis, and promoted recovery of motor performance in SCI rats [82]. Ansorena et al. found that GDNF-loaded injectable alginate hydrogels stimulated neurite growth and functional recovery after SCI with more growing neuritis at the lesion site [83]. Additionally, platelet-derived growth factor (PDGF) promoted the differentiation of NSCs into neuronal cells, ECM synthesis, and angiogenesis [100]. Wu et al. indicated that supramolecular hydrogel microspheres of PDGF promoted the recovery of SCI by inhibiting M1 macrophage infiltration and extrinsic or intrinsic cells apoptosis, promoting the survival of NSCs and neuronal differentiation, and stimulating synapse formation and angiogenesis in SCI rats [84]. Additionally, a novel injectable Lap/Hep hydrogel containing FGF4 exhibited strong neuroprotection and regeneration after SCI by inhibiting inflammatory response, increasing myelination regeneration and reducing glial/fibrotic scarring [85]. However, the molecular mechanisms involved in certain GF-induced neural repair need to be elucidated in depth.

## 4. Therapeutic Mechanism of Injectable Hydrogels in SCI

The current injectable hydrogels are mainly carried out from several aspects, such as anti-inflammatory, antioxidant, anti-apoptosis and pro-neurogenesis when repairing SCI (Table 3).

### 4.1. Anti-Inflammation

The inflammatory response following SCI is a complex process coordinated by many cell types and inflammatory factors, including tumor necrosis factor-α (TNF-α), interleukin-1β (IL-1β), interleukin-6 (IL-6), interferon-γ (IFN-γ), etc. [122]. TNF-α and IL-6 are significantly upregulated around the area of SCI from 3 to 24 h [5]. Although inflammation is a universal consequence of systemic trauma and an essential defense mechanism for the host, ref. [123] inflammation in SCI is a double-edged sword [124]. For one thing, the inflammatory response is necessary to effectively remove tissue debris and promote wound healing and tissue repair. For another, various factors harmful to neurons, glial cells, axons, and myelin are also released during the inflammatory response. With the increase of inflammatory cytokines, the toxic microenvironment leads to the formation of cavities and glial scars, thus inhibiting the recovery of nerve function [125]. Microglia/macrophage-mediated neuroinflammation persists for a long period of time and affects SCI repair. Therefore, suppression of chronic inflammation is favorable for the recovery of SCI, but the timing of inflammatory interventions should be kept in mind [126]. Hydrogel can control the release of stem cells, anti-inflammatory drugs, GFs, etc., and improving the local microenvironment in the lesion, which has broad application prospects in SCI treatment [127].

Xin et al. designed a hydrogel loaded with bazedoxifene (BZA, an anti-inflammatory agent) based on HA, sodium alginate (SA), and polyvinyl alcohol (PVA) to effectively deliver BZA to the site of SCI, which significantly reduced inflammation in the lesion of SCI and attenuated the disruption of the blood–spinal cord barrier, which was mediated by the regulation of the NF-κB/MMP signaling pathway [101]. Additionally, electroconductive gelatin hydrogels loaded with bone marrow derived mesenchymal stem cells (BMSC)-exosomes synergistically promoted tissue repair after SCI by regulating microglial M2 polarization, modulating inflammation and enhancing myelinated axon growth via the NF-κB pathway [102]. Xu et al. revealed that a biocompatible HAMC (hyaluronan and MC) hydrogel loaded with fat extract significantly inhibited the death of neuronal cells and modulated the inflammatory phenotype of macrophages in the locally injured region of SCI [103]. In addition, PLGA-PEG-PLGA thermosensitive hydrogels containing baricitinib inhibited the expression of the JAK2-STAT3 pathway and inflammatory cytokines in the early stages of injury, reduced neuronal apoptosis, and promoted functional recovery in rats with SCI [80]. Furthermore, in situ heparin hydrogel injection containing bFGF and dental pulp stem cells (DPSCs) prevented microglia/macrophage activation and reduced proinflammatory cytokine release, which is benefit to nerve regeneration after SCI [104].

Han et al. created HA hydrogels containing tauroursodeoxycholic acid inhibited the inflammatory effect and promoted functional recovery after SCI by reducing pro-inflammatory cytokine (IL-1β, IL-6, IFN-γ and TNF-α) levels [105]. PCL nanofiber-modified hydrogel consisting of HA and poly(ethylene glycol) diacrylate promoted macrophage polarization in a rat SCI model, leading to the enhanced immature neuron amount and axon density [106]. The hydrogels prepared by Sun et al. regulated inflammation by modulating microglia/macrophages and can block chondroitin sulfate proteoglycan (CSPGs) inhibitory signaling receptors to promote an anti-inflammatory phenotype [107]. In conclusion, composite hydrogels can reduce inflammatory response, improve the local microenvironment, and accelerate the treatment process during SCI treatment. However, most of these studies have been performed on rodent models. It is urgent to determine the critical timeline for cytokine release following SCI and to further explore the optimal timing of hydrogel treatment for SCI.

### 4.2. Antioxidant

SCI is accompanied by the loss of ionic homeostasis, glutamate excitotoxicity, mitochondrial dysfunction, and oxidative stress [88]. The accumulation of large amounts of reactive oxygen species (ROS) leads to massive neuronal death, which further leads to secondary damage in SCI. Inhibition of post-injury peroxidation of biomolecules through effective antioxidant interventions will be a strategy for the treatment of SCI [128]. Therefore, functionalized hydrogels with free radical scavenging capacity or loaded with antioxidants will be beneficial for SCI recovery and functional reconstitution [129].

For example, the manganese-dioxide-nanoparticle-dotted (MnO_2_NPs) HA hydrogel prepared by Li et al. regulated the ROS microenvironment of SCI, thereby effectively improving the viability of MSCs and synergistic promotion of spinal cord repair [108]. Liu et al. prepared a N-acryloylglycinamide/methacrylic gelatin/laponite/tannic acid (TA) hydrogel combined with MSC-derived small extracellular vesicles (MSC-sEVs), which can realize local, sustainable, and stable delivery of MSC-sEVs at the SCI site, effectively scavenge free radicals, and reduce the expression of 4-hydroxynonenal and 8-hydroxydeoxyguanosine caused by oxidative stress [109]. Chen et al. constructed polydopamine-modified hydrogel coating exosomes derived from sophora, and this composite hydrogel rapidly improved impaired motor function and relieved voiding dysfunction by modulating the oxidative stress microenvironment [110]. In addition, a BMSC-encapsulated ROS-scavenging hydrogel synthesized by methacrylate HA, IKVAV peptides, and growth factors (EGF and bFGF), significantly alleviated oxidation, inflammation, and cell apoptosis, resulting in better neurogenesis and motor recovery, accompanied by attenuation of scar formation in a rat model of SCI [111]. Liu et al. constructed a gelatin-modified hydrogel laden with NSCs and albumin-incubated CeO2 nanoparticles, which could promote neurogenesis via alleviating oxidative stress microenvironments and improving the viability of encapsulated NSCs [112]. The hydrogel prepared by Du et al. via polymerization of α-lipoic acid had inherent antioxidant capacity and can effectively provide wound-healing and spinal cord injury treatment, removing ROS from the injury site [113]. Thus, the antioxidant function of injectable hydrogels provides a new therapeutic idea for the treatment of SCI. Nevertheless, it is still a challenge to develop bioactive scaffolds with excellent outstanding antioxidant capacities and outstanding biocompatibilities for SCI therapy.

### 4.3. Anti-Apoptosis

Apoptosis is a physiological process that occurs in cell development, but damaged cells die during apoptosis [130]. There are two pathways of cell death in the injured spinal cord: immediate necrosis and delayed apoptosis of cells. The latter lasts for approximately 14 days after trauma and involves neurons and glial cells that are far from the traumatic area [131]. Apoptosis may lead to neuronal cell death and play an important role in the pathogenesis of neurological disorders [132]. The main genes involved in apoptosis are Bcl-2 (apoptosis inhibitor) and Bax (apoptosis promoter). A growing number of studies have shown that injectable hydrogels can reduce neuronal apoptosis and promote neuronal cell survival, which has great potential in SCI treatment [133].

For example, Yuan et al. developed a CaNeu hydrogel as a delivery vehicle for ADSCs, and studies have shown that this hydrogel significantly inhibited neuroinflammation and cell apoptosis by reducing the expression of the pro-apoptotic protein Bax at the lesion site, while increasing the expression level of anti-apoptotic protein Bcl-6 [76]. Li et al. co-immobilized umbilical cord MSCs and bFGF in ECM and HP to form a bioactive, heat-sensitive hydrogel, which exerted promising utility for the functional recovery of SCI by reducing cell apoptosis and improving mitochondrial function [114]. In addition, Wu et al. produced PDGF mimetic peptide hydrogel microspheres to encapsulate NSCs, which significantly inhibited M1 macrophage infiltration and extrinsic or intrinsic cells apoptosis on the seventh day after SCI [84]. Similarly, gelatin methacryloyl (GelMA) hydrogel implants loaded with activated Schwann cells obviously inhibited cell apoptosis and promoted functional recovery following SCI [115]. Alginic acid sodium hydrogel co-transplantation with Schwann cells inhibited cellular apoptosis, enhanced Bcl-2 expression, and thereby promoted the recovery of locomotor function after SCI [116]. In addition, hydrogels loaded with zinc oxide nanoparticles could increase the production of SOD, GSH, Nrf2 and HO-1 at the site of injury and downregulate ROS intensity when used in combination therapy for spinal cord transection [117]. In short, injectable hydrogels can reduce neuronal apoptosis at lesions of SCI and are beneficial to the treatment of SCI. Meanwhile, the molecular mechanisms by which complex hydrogels anti-apoptosis after SCI still need to be further explored, e.g., whether the effects are direct or indirect and the target molecules involved.

### 4.4. Pro-Neurogenesis

Severe and chronic SCI are often associated with the permanent loss of neurological function, mainly due to the failure of injured axons to regenerate and rebuild functional connections and the loss of neurons. Therefore, promoting neural regeneration is a feasible idea for improving sensorimotor recovery of SCI [2].

Neural regeneration is the regeneration and repair of damaged neural tissue (neurons, axons, synapses, and glial cells) after injury [134], which includes the elongation of axons, the germination and growth of new axons, or the regeneration of neuronal cells [135]. Thus, regeneration, including both neuronal and axonal regeneration, is a complex biological process that requires joint coordination [136]. Current drug- or cell-based SCI therapies fail to provide topographic guidance for regenerating neurons and result in random growth and poor therapeutic efficacy [134]. Injectable hydrogels as biological scaffolds can not only load drugs or cells but can also create structures that allow neuronal growth and guide axon regeneration throughout the injury site [137].

Axons are the tiny nerve fibers that connect neurons and allow them to communicate [138]. Zhang et al. found that GelMA hydrogel lengthened the axons of mouse neurons, increased the expression of growth-related protein GAP43, and promoted the recovery of neurological function of SCI mice [118]. Fan et al. demonstrated that gelatin methacrylate (GM)-modified hydrogels immobilized BMSC exosomes and promoted axon outgrowth and neural synaptic network formation in vitro. Additionally, this hydrogel induced endogenous NSCs recruitment, enhanced neuronal regeneration, and inhibited astrocytic proliferation, providing a favorable microenvironment for later axonal regeneration in SCI mice [102]. Yao et al. found that MSC-laden fibrin hydrogel implantation enhanced the donor MSC neural differentiation, encouraged the migration of host neurons into the injury gap, and significantly promoted nerve fiber regeneration [119]. In addition, anti-inflammatory peptides and BDNF-modified hyaluronic acid-methylcellulose (HAMC) hydrogels inhibited local inflammation and promoted neuronal survival as well as axon regeneration in SCI rats [120]. Furthermore, Agarwal et al. developed a conductive graphene cross-linked collagen (Gr-Col) cryogel, which could promote axonal regeneration by suppressing astrocyte reactivity and modulating microglial polarization in SCI rats [121]. Hence, these data suggest that injectable hydrogels loaded with drugs/cells or GFs can promote neural regeneration and SCI repair. Although significant efforts have contributed to exploring neurogenesis-based strategies for treating SCI, there are still significant knowledge gaps. After all, neural regeneration-especially axonal regeneration—is a complex process regulated by multiple factors, but the mechanisms regulating regeneration should be illustrated in depth.

## 5. Combination Therapy

Currently, low-frequency pulsed electromagnetic field (LFPEMF) is a clinically used non-invasive therapeutic measure for neural repair that has been shown to prevent inflammation and oxidative stress, and it exhibits powerful neuroprotective effects in the nervous system [139]. Conductive hydrogels are attractive candidates for accelerating SCI repair because they match the electrical and mechanical properties of the neural tissues [140]. Therefore, hydrogels combined with electromagnetic stimulation (ES) to treat SCI have become an interesting strategy. For example, Liu et al. demonstrated that combined with ES by electrode needles, thermosensitive-electroactive-hydrogel-loaded NGF significantly inhibited astrocyte differentiation and restored spinal circuitry and locomotor function by stimulating endogenous neurogenesis in a rat SCI model [141]. Moreover, implantation of an IONP-embedded gelatin–genipin hydrogel system along with MF (17.96 μT, 50 Hz uniform EMF) exposure modulated the microenvironment, making it conducive to neural repair and regeneration after SCI in rats [142]. Furthermore, He et al. indicated that ES promoted axon outgrowth and Schwann cell migration away from dorsal root ganglia spheres cultured on the hybrid hydrogel incorporated pristine carbon nanotubes [143]. HA/collagen hydrogels loaded with Fe_3_O_4_@BaTiO_3_ NPs, stimulated by an external pulsed magnetic field, are able to enhance nerve regeneration both at the cellular level and at SCI mice [144]. Thus, hydrogels combined with ES are beneficial to nerve regeneration in SCI repair. Though there are not many relevant studies, their advantages make this method very promising.

Additionally, phototherapy is also a promising strategy for the treatment of SCI [145]. The combination of hydrogels and phototherapy is more effective for treating SCI, because phototherapy could further promote hydrogels to mimic ECM and improve their therapeutic efficacy. Cai et al. constructed macroporous functional hydrogels (MFH) with guide catheters to encapsulate photosensitive phenyl azides and proteins. They found that photoimmobilization of collagen significantly improved the adhesion and survival of NSCs in the catheter and that the optimized hydrogel scaffolds improved motor recovery in rats 12 weeks after SCI [146].

## 6. Conclusions and Prospects

**Figure 3:**
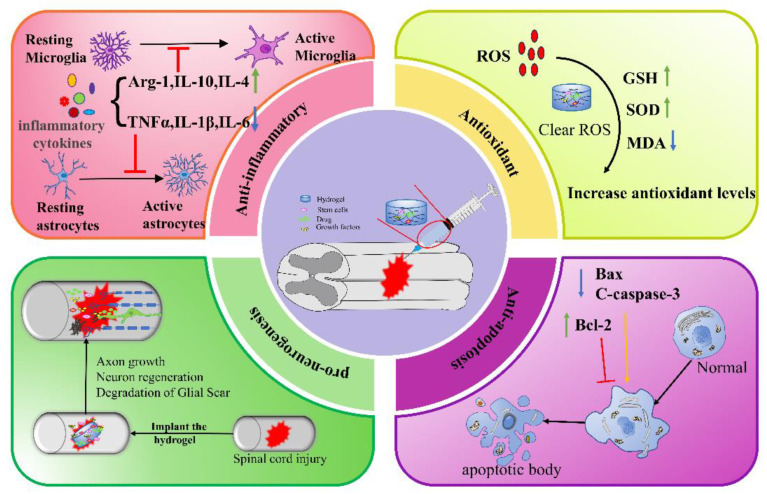
Mechanisms of action of injectable hydrogel in the treatment of spinal cord injury. Green arrow: up; blue arrow: down; yellow arrow: facilitation; red arrow: inhibition.

In summary, natural, synthetic, and composite injectable hydrogels can all be used as delivery systems to encapsulate stem cells, drugs, or GFs for a wide range of applications in SCI therapy. The mechanisms by which hydrogels promote SCI repair include anti-inflammation, anti-oxidation, anti-apoptosis, and pro-neurogenesis (Figure 3), etc. In addition, hydrogel combined with electromagnetic stimulation or phototherapy can also improve the repair of SCI. Although much progress has been made in the study of injectable hydrogels for SCI, there are still certain limitations. Firstly, the toxicity and swelling of the polymers used to synthesize hydrogels may have a detrimental effect on SCI treatment. Therefore, there is a need to find new and better biocompatible biomaterials. Secondly, the modalities and time windows of hydrogel therapy need more exploration. It is urgent to improve the injection conditions when implanting at the site of injury and to ensure that the properties of the hydrogel are matched to the implantation site. Thirdly, the translation from preclinical models to the clinical setting is challenging. Despite a large number of studies in preclinical SCI models, few have been successful in clinical trials. We need to optimize conditions, conduct preclinical studies in primate models, and ultimately transfer the performing strategies to clinical trials. On the other hand, if we can find more compatible synthetic materials, actively optimize the synthesis method of hydrogels, and combine hydrogel transplantation with other therapeutic approaches to overcome the shortcomings of hydrogels in terms of cellular and biotoxicity, hydrogels will play an active role in the treatment of many diseases. Regardless, hydrogels as delivery systems offer alternative treatment options for SCI therapy and are an area of ongoing breakthroughs with a wide range of applications.

## Figures and Tables

**Table 1:** Classification and application of hydrogels.

Hydrogels	Materials	Models	Function	Ref
Natural hydrogel	Hyaluronic acid (HA)	Hemisection spinal cord injury/Laminectomy	Reduce inflammation; promote angiogenesis and myelination; limit astrocyte activation	[28,29,30]
	Collagen	Laminectomy results in complete spinal cord transection	Promote neurogenesis, inhibit cell apoptosis and reduce glial scars production	[31,32,33]
	Chitosan	Complete spinal cord transection	Inhibit neuroinflammation; promote the recovery of motor function	[34,35,36,37]
	Alginate	Hemimyelonectomy of 2 mm or 4 mm length/complete spinal cord transection	Promote spinal cord neural stem/progenitor cell differentiation and locomotor recovery; promote axonal growth	[38,39,40]
	Agarose	Hemisection spinal cord injury	Promote nerve regeneration after SCI	[41]
Synthetic hydrogel	Polyethylene glycol (PEG)	Dorsal hemisection lesion model/Complete transection model/Spinal cord re-transection at 8 months post-resection	Promote axon regeneration, myelination	[42]
	Polyacrylamide (PAM)	Spinal cord cells of SD rats	Improve the motor function	[43]
	Polyhydroxyethyl methacrylate (PHEMA)	Complete spinal transection at the T9 level; Spinal cord hemisection model	Promote neurite growth and the production of blood vessels	[44,45,46]
	poly-ε-caprolactone (PCL)	SCI model of the T9 dorsal hemisection	Restore the continuity of the damaged spinal cord and decreased cavity formation	[47,48]
Composite hydrogel	Gelatin-acrylated-β-cyclodextrin-polyethene glycol diacrylate	SCI model of the right hemiesection of the spinal cord	Induce nerve regeneration and functional recovery	[49]
	Fibrin and functionalized self-assembling peptides	Lateral hemisected SCI model	Accelerate axonal regeneration Promote angiogenesis	[50]
	HA, methylcellulose (MC), and polylactic acid-glycolic acid (PLGA)microparticles	Complete spinal cord injury	Achieve continuous and topical delivery of therapeutic drugs	[51]

**Table 2:** Application of hydrogels as delivery systems in SCI.

Encapsulated Substances	Hydrogel Composition	Specific Substances	Function	Ref
Stem cells	PLGA; TA; oxidized dextran (Dex) and hyaluronic acid-hydrazide	NSCs	Promote the differentiation of NSCs into neurons while inhibiting the differentiation of astrocytes	[72]
	HA; collagen	NSCs	Promote NSCs to differentiate into neuron-like cells and play neuroprotective roles	[73]
	Gelatin; peroxidase (HRP) and galactose oxidase (GalOx)	MSCs	Enhance the neural differentiation and functional recovery of SCI;	[74]
	ECM	Human iPSCs	Reduces inflammation, enhance nerve regeneration, and significantly improve movement	[75]
	Gelatin; methacrylic	ADSCs	Promote axon growth, reduce neuroinflammation, and ultimately improve motor function in SCI rats	[76]
Drugs	Alginate; chitosan	Erythropoietin (EPO)	Improve tissue repair and histopathological appearance of the spinal cord at the site of injury	[77]
	Chitosan; collagen	Serpins	Improve the neurological and motor function and reduce tissue damage caused by in-flammation in SCI rats	[78]
	Chitosan	Cannabidiol (CBD)	Reduce apoptosis, improve neurogenesis by enhancing mitochondrial biogenesis	[79]
	PLGA; PEG	Baricitinib	Reduce neuronal apoptosis and promote functional recovery in SCI rats	[80]
	Chitosan	Curcumin	Favor functional recovery of SCI rats	[81]
Growth factors	Heparin-poloxamer	bFGF and NGF	Improve neuronal survival, inhibit reactive astrogliosis, and promote recovery of motor performance	[82]
	Alginate	GDNF	Stimulate neurite growth and functional recovery	[83]
	Naphthalene acetic acid-phenylalanine-phenylalanine-glycine	Platelet-derived growth factor (PDGF)	Inhibit M1 macrophage infiltration and extrinsic or intrinsic cells apoptosis	[84]
	Heparin-Laponite	FGF4	Inhibit inflammatory response, increase myelination regeneration, and reduce glial/fibrotic scarring	[85]

**Table 3:** Therapeutic mechanism of injectable hydrogels in SCI.

Therapeutic Mechanisms	Hydrogel Composition	Animal Models	Specific Performance	Ref
Anti-inflammation	HA; sodium alginate (SA); polyvinyl alcohol (PVA)	SCI model caused by impact injuries	Reduce inflammatory cell infiltration	[101]
	Gelatin methacrylate (GM); Polypyrrole (PPy); tannic acid (TA)	Longitudinal right spinal cord hemisection	Promote M2 microglial polarization	[102]
	Hyaluronan; MC	Model of spinal cord contusion	Promote the polarization of macrophages	[103]
	PLGA; PEG; baricitinib	Acute spinal cord injury	Inhibit M1 polarization	[80]
	bFGF; dental pulp stem cells (DPSCs); heparin	Clamp a vascular clip on the spinal cord tissue at T9 for 2 min	Prevent microglia activation	[104]
	Taurinedeoxycholic acid; HA	SCI caused by descent impact	Reduce levels of pro-inflammatory cytokines	[105]
	Polyethylene glycol diacrylate; HA	SCI caused by impact injuries	Facilitate polarization towards the M2	[106]
	intracellular sigma peptide (ISP); intracellular LAR peptide (ILP); Chondroitinase ABC (ChABC)	Complete transection SCI model	Secret anti-inflammatory cytokines	[107]
Antioxidant	MnO_2_NPs; HA	Complete transection SCI model	Reduce intracellular ROS levels	[108]
	N-acryloylglycinamide/methacrylic gelatin/laponite/TA	Complete transection model of the spinal cord	Scavenge free radicals and reduce 4-hydroxynonenal expression	[109]
	Polydopamine; HA	Complete transection model of the spinal cord	Reduce ROS levels	[110]
	ROS-responsive hyperbranched polymers; methacrylate hyaluronic acid	Complete transection model of the spinal cord	Reduce endogenous ROS and oxidative damage	[111]
	gelatin methacryloyl (GelMA); cerium oxide; NSCs	Complete transection model of the spinal cord	Reduce lipid peroxidation	[112]
	α-Lipoic acid (LA)	SCI caused by impact injuries	Clear ROS	[113]
Anti-apoptosis	Dimethylformamide; β-CD; Acryloyl Chloride	Complete transection model of the spinal cord	Reduce the expression of Bax	[76]
	bFGF; ECM; heparin-poloxamer (HP)	SCI model caused by impact injuries	Reduce apoptosis; Improve mitochondrial function	[114]
	Platelet-derived growth factor (PDGF); melamine phosphate	SCI model caused by miniature tweezer clamping	Inhibit exogenous or intrinsic apoptosis	[84]
	GelMA	Spinal cord right semitransection model	Decrease caspase-3 expression and increase Bcl-2 expression	[115]
	SA	Complete transection model of the spinal cord	Inhibit apoptosis and enhance Bcl-2 expression	[116]
	Zinc oxide nanoparticles (ZnONPs); hyaluronic acid (HA)	SCI model caused by impact injuries	Improve the production of SOD, GSH, Nrf2, and HO-1	[117]
Pro-neurogenesis	Gelatin; methacrylic anhydride	Clamp the mice spinal cord tissue using an aneurysm clip	Lengthen mouse neuronal axons	[118]
	Methylacrylic anhydride; gelatin; pyrrole	Spinal cord right semitransection model	Promote axon growth and neuronal regeneration	[102]
	Fibrin; MSCs	Long-distance spinal cord transection injury	Promote nerve fiber and axons regeneration	[119]
	HA; MC	Clamp the mice spinal cord tissue using an aneurysm clip	Promote neuronal survival and axon regeneration	[120]
	Collagen; graphene	Rat spinal cord lateral hemisection model of SCI	Promote myelination and increase expression of mature axon markers	[121]

## Data Availability

Not applicable.

## Abbreviations

SCI: Spinal cord injury; EPO, erythropoietin; ESCs, embryonic stem cells; iPSCs, pluripotent stem cells; MSCs, mesenchymal stem cells; NSCs, neural stem cells; HA, hyaluronic acid; ECM, extracellular matrix; SD, Sprague-Dawley; PEG, Polyethylene glycol; PAM, polyacrylamide; PHEMA, Polyhydroxyethyl methacrylate; PCL, poly-ε-caprolactone; GFs, growth factors; MC, methylcellulose; PLGA, polylactic acid-glycolic acid; CaNeu, cell adaptive neurogenic; ADSCs, adipose-derived stem cells; CBD, Cannabidiol; JAK, Janus kinase; bFGF, fibroblast growth factor; BDNF, brain-derived neuotrophyic factor; NGF, nerve growth factor; HP, heparin-poloxamer; PDGF, platelet-derived growth factor; TNF-α, tumor necrosis factor-α; IL-1β, interleukin-1β; IL-6, interleukin-6; IFN-γ, interferon-γ; SA, sodium alginate; BMSC, bone marrow derived mesenchymal stem cells; ROS, reactive oxygen species; TA, tannic aci; GelMA, gelatin methacryloyl; ES, electromagnetic stimulation.
